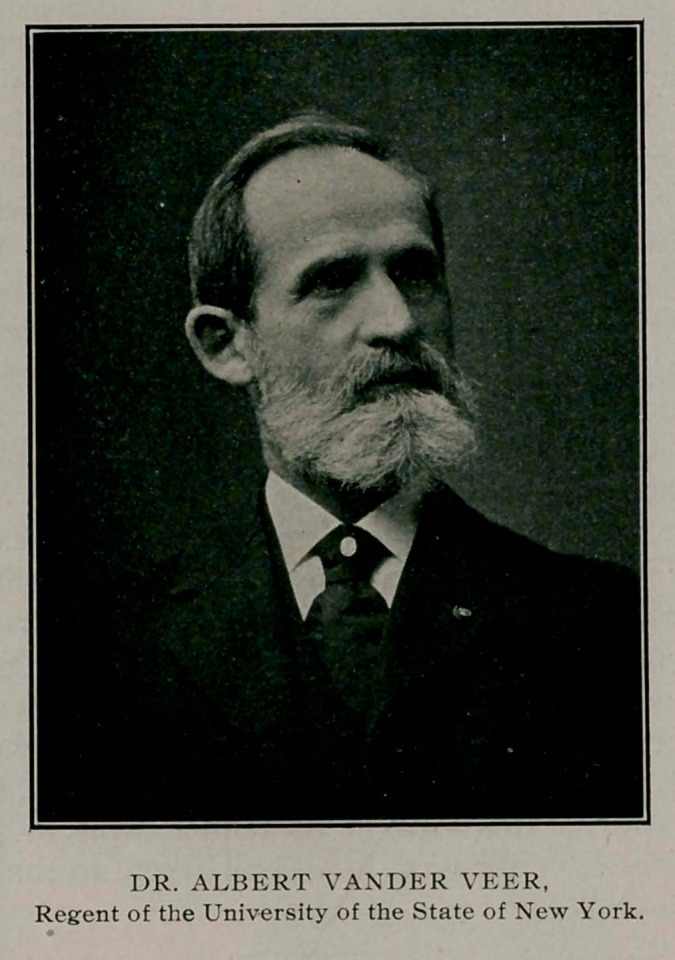# Regent Vander Veer

**Published:** 1906-05

**Authors:** 


### Regent Vander Veer

Dr. Albert Vander Veer, of Albany, was elected regent of the University of the State of New York by the legislature, on Wednesday, April 25, 1906, to fill the unexpired term of Regent

Charles S. Francis, of Troy, resigned. Mr. Francis has been appointed Ambassador to the Austria-Hungary Court, hence his resignation as regent. His term would expire in 1915, therefore Dr. Vander Veer will serve nine years.

It may not be know generally that regents of the University are elected in the same manner in which United States senators are chosen. It is customary for the members of each party to hold a caucus the night before the election, to select the candidates to be voted for the next day. On Tuesday evening, April

DR. ALBERT VANDER VEER,
Regent of the University of the State of New York.

24, the republicans of both houses agreed to support Dr. Vander Veer, and the democrats selected Mr. Charles E. Patterson as their candidate. The next day at noon the election was held with the result already mentioned.

Dr. Vander Veer has already served ten years as regent, and therefore brings a valuable experience with him as he enters the board again. He was first elected in 1895. When the number of regents was reduced to eleven by the law of 1904 Regent Vander Veer was retained, and in the drawing of lots for terms he drew the short straw—one year. Upon the expiration of his term it was determined that he was ineligible for reelection because the law prohibited the choice of two regents from the same judicial district. Mr. Francis, of Troy, who drew the long term, two years ago, resides in the third district, and now retires for the reason stated, leaving a vacancy to which the legislature with wisdom and justice elects former Regent Vander Veer.

Dr. Vander Veer is one of the most famous surgeons of the United States and is the reigning president of the American Surgical Association. He is also dean and professor of surgery in Albany Medical College,—the medical department of Union University. He is the author of many valuable brochures and monographs on surgical subjects and upon medical education. He was one of the committee of conference on the Amalgamation of the Medical Society of the State of New York and the New York State Medical Association, so happily accomplished last winter.

Regent Vander Veer will bring a ripened experience to the board of regents to which he returns, and will be a wise counsellor regarding the medical affairs with which the regents often have to deal. The medical profession is to be congratulated upon this excellent choice of the legislature.

---

A year ago, that is to say in the May 1905 edition of this Journal, we made reference to paper milk bottles as a prospective hygienic reform and gave a detailed description of the new milk container. It appears, according to the *New York Tribune,* that we are near the realisation of expectation in this regard, as indicated by the following paragraph, which that paper printed April 21:

Paper milk bottles, which were promised to the public a year or two ago, are likely to appear in the market almost any day. Their great merit is that they cannot be used a second time. Hence, neglect to wash them properly cannot be attended with the consequences which often follow similar treatment of glass bottles. There is talk, too, of manufacturing beer and soda water bottles from the same material. Whether these would have any particular sanitary value may, perhaps, be questioned, but refilling a receptacle with an imitation of the original contents would be effectually prevented.

---

The Regents of the University of the State of New York, at a meeting held at Albany, April 26, 1906, made the following-mentioned appointments of State Medical Examiners:

From the State Medical Society: Dr. William Warren Potter of Buffalo; Dr. William S. Ely of Rochester, and Dr. Maurice J. Lewi of New York City, all reappointed for the term of three years from August 1, 1906; and Dr. Arthur W. Booth of Elmira to fill the unexpired term of Dr. George Ryerson Fowler, deceased.

From the Homeopathic Medical Society: Dr. J. M. Lee of Rochester; Dr. J. W. Candee of Syracuse and Dr. G. E. Gorham of Albany, all reappointed for three years, from August 1, 1906.

From the State Eclectic Society: Dr. L. H. Smith of Buffalo; Dr. O. W. Sutton of Bath and Dr. M. H. Nichols of Worcester; all reappointed for three years, from August 1, 1906. Anna L. Alline of New York City was appointed a member of the state board of nurses' examiners.

The next convocation of the state university will be held from October 26 to October 28, instead of in June, as formerly. The change is made to have more college and secondary school men in attendance.

The board decided that no city union free school district or academy shall share in the apportionment of school moneys unless its schools participate in the academic examinations as prescribed by the board.

## Figures and Tables

**Figure f1:**